# Expression and Role of GPR87 in Urothelial Carcinoma of the Bladder

**DOI:** 10.3390/ijms140612367

**Published:** 2013-06-10

**Authors:** Homare Okazoe, Xia Zhang, Dage Liu, Shinsuke Shibuya, Nobufumi Ueda, Mikio Sugimoto, Yoshiyuki Kakehi

**Affiliations:** 1 Department of Urology, Kagawa University Faculty of Medicine, 1750-1 Ikenobe, Miki-cho, Kita-gun, Kagawa 761-0793, Japan; E-Mails: okazoe@me.com (H.O.); zhangxia@med.kagawa-u.ac.jp (X.Z.); nob@med.kagawa-u.ac.jp (N.U.); micsugi@med.kagawa-u.ac.jp (M.S.); 2 Department of General Thoracic Surgery, Kagawa University Faculty of Medicine, 1750-1 Ikenobe, Miki-cho, Kita-gun, Kagawa 761-0793, Japan; E-Mail: dgliu@med.kagawa-u.ac.jp; 3 Department of Diagnostic Pathology, Kagawa University Faculty of Medicine, 1750-1 Ikenobe, Miki-cho, Kita-gun, Kagawa 761-0793, Japan; E-Mail: sshibuya@med.kagawa-u.ac.jp

**Keywords:** GPR87, non-muscle-invasive bladder cancer, intravesical recurrence, progression

## Abstract

The orphan GPR87 has recently been matched with its ligand LPA, which is a lipid mediator with multiple physiological functions, including cancer cell proliferation. This study aimed to clarify the role of GPR87 in urothelial carcinoma of the bladder. GPR87 expression was assessed in seven human bladder cancer cell lines. A replication-deficient recombinant adenoviral vector expressing shRNA targeting GPR87 (Ad-shGPR87), was constructed. Gene silencing was carried out using Ad-shGPR87. Immunohistochemical analysis was performed for transurethral resection of bladder tumor samples from 71 patients with non-muscle-invasive bladder cancer. We observed GPR87 expression in five of the seven cell lines, and silencing GPR87 gene expression significantly reduced cell viability. GPR87 expression was positive in 38 (54%) of 71 tumors. Ki-67 index was associated with positive GPR87 staining status (*p* < 0.0001). Patients with GPR87-positive tumors had shorter intravesical recurrence-free survival than those with GPR87-negative tumors (*p* = 0.010). Multivariate analysis revealed that GPR87 staining status was an independent prognostic parameter for intravesical recurrence (*p* = 0.041). Progression from non-muscle-invasive to muscle-invasive tumor was more frequently observed in patients with GPR87-positive tumors, although this trend did not reach statistical significance (*p* = 0.056). These results warrant further prospective studies to clarify the role of GPR87 expression in intravesical recurrence and progression in bladder cancer.

## 1. Introduction

GPCRs constitute the largest family of transmembrane receptors. To date, over 800 members have been cloned, from a wide range of species. All have a similar characteristic motif consisting of seven distinct hydrophobic regions that is regarded as the transmembrane domain. Certain GPCRs have already been identified as responsible for the signal transduction of a diverse variety of ligands, including nucleotides, biogenic amines, peptides, and other small molecules. However, approximately 150 of the cloned GPCRs have remained as orphan receptors with unknown ligands. GPCRs are assumed to be of particular importance in cancer biology because several GPCR ligands, such as bioactive peptides, biogenic amines, and chemokines, are highly expressed in the tumor microenvironment. Thus, GPCR signaling promotes tumor cell growth and survival, angiogenesis, metastasis, and drug resistance [1–4].

In 2001, Lee *et al.* discovered the orphan GPR87, also known as GPR95, together with nine other GPCRs by performing customized searches of the GenBank high-throughput genomic sequences database with previously known GPCR-encoding sequences [5]. In 2007, GPR87 was deorphanized and shown to be a lysophosphatidic acid (LPA) receptor [6]. It is particularly interesting that LPA is an extracellular bioactive phospholipid that mediates diverse biological activities, including cancer cell proliferation, invasion, and angiogenesis [7]. Prior to the identification of GPR87, three LPA receptors (LPA1, LPA2, LPA3) had been identified. We previously reported that LPA3 (also known as Edg7) was deeply involved in prostate cancer development and progression [8]. A comprehensive analysis using gene chip technology demonstrated that GPR87 mRNA was preferentially overexpressed in squamous cell carcinoma at different locations. In addition, GPR87 was overexpressed in some bladder cancer tissues and adenocarcinoma of the lung [9,10].

In the present study, we measured GPR87 mRNA expression levels in several bladder cancer cell lines and investigated the influence of silencing GPR87 mRNA on cell proliferation. Protein expression levels of GPR87 in surgical bladder cancer specimens were also immunohistochemically analyzed, and special attention was paid to cell proliferation, including tumor grade and Ki-67 index. The relationship of GPR87 expression with intravesical recurrence and progression of non-muscle-invasive bladder cancer was also investigated.

## 2. Results and Discussion

### 2.1. GPR87 Expression in Bladder Cancer Cell Lines

**Figure 1:**
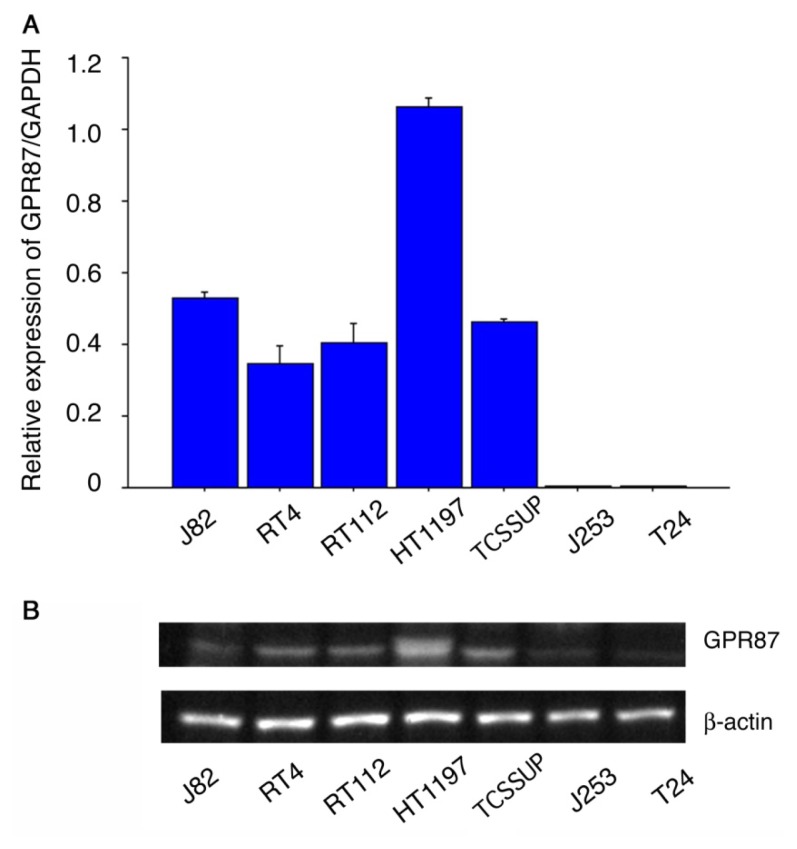
GPR87 expression in seven human bladder cancer cell lines. Relative expression levels of GPR87/GAPDH mRNA assessed by real-time RT-PCR (**A**) and GPR87 protein expression assessed by western blots (**B**).

Figure 1A shows GPR87 gene expression relative to GAPDH expression in seven human bladder cancer cell lines. In five (HT1197, J82, TT112, RT4 and TCCSUP) cell lines, GPR87 mRNA levels were substantial, whereas the GPR87 mRNA levels were very low in the J253 and T24 cell lines. Western blots of cell lysates from the seven cell lines revealed that protein expression was comparable to gene expression, as shown in Figure 1B.

### 2.2. GPR87 Knockdown

**Figure 2:**
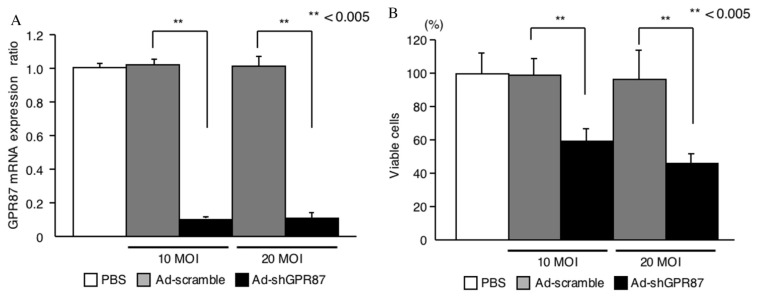
Suppressive effect of Ad-shGPR87 on GPR87 gene expression (**A**) and cellular viability (**B**) in human bladder cancer HT1197 cells. The error bars indicate standard deviations.

Because GRP87/GAPDH mRNA was most highly expressed in the HT1197 cell line, it was subjected to the following gene silencing experiment. GPR87 mRNA level was suppressed following transfection of Ad-shGPR87 in a time-dependent manner until 120 h. Suppression of GPR87 expression was evaluated using Ad-shGPR87 at infection (MOI, PFU/cell) of 10 and 20 (Figure 2A). Cellular viability was assessed 120 h after transfection of Ad-shGPR87, and significant reduction in a dose-dependent manner (55% at 10 MOI; 41% at 20 MOI respectively) was observed compared to control cells transfected with Ad-scramble, as shown in Figure 2B.

### 2.3. GPR87 Expression in Tumors

#### 2.3.1. Association with Proliferation

**Figure 3:**
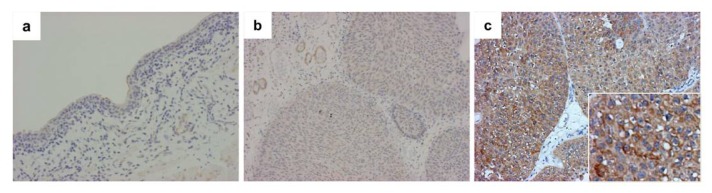
Immunostaining for GPR87. The normal mucosa of the bladder is negative for GPR87 (**a**). A representative negative staining (**b**) and positive staining in non-muscle-invasive bladder cancers (**c**) are shown.

**Figure 4:**
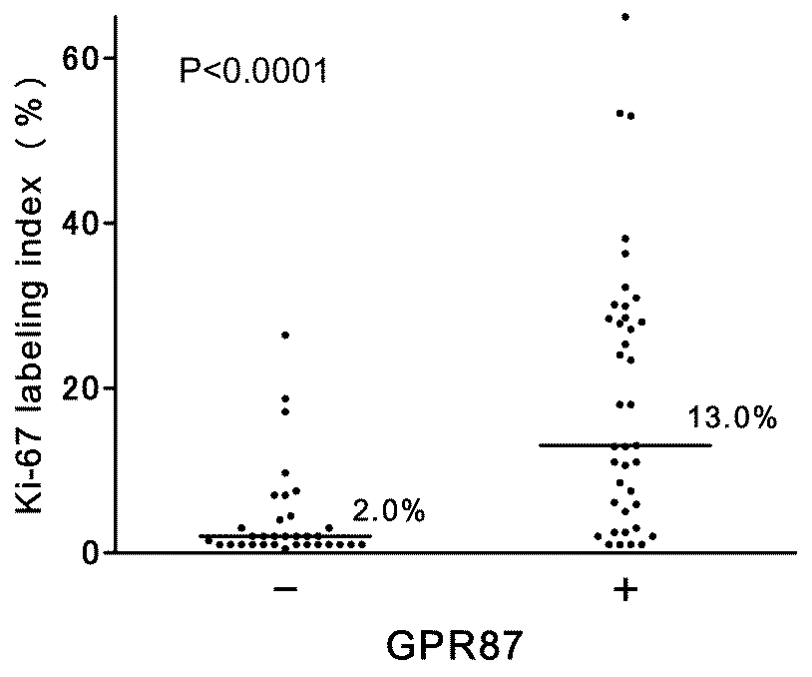
Relationship between GPR87 expression status and Ki-67 labeling index in non-muscle-invasive bladder cancers.

In urothelial tumor cells, GPR87 immunostaining was positive in 38 (54%) of 71 non-muscle-invasive bladder cancers. In positive-staining cases, GPR87 was predominantly found in cancer cell membranes, but the cytoplasm was sometimes also stained, as shown in Figure 3. The positive-staining ratio in high-grade tumors was higher than that in low-grade tumors (16 of 25 *vs.* 22 of 46), although the difference was not statistically significant (*p* = 0.136). However, there was a strong correlation of GPR87 expression with proliferative activity. The median Ki-67 index in GPR87-positive tumors was higher than that in GPR87-negative tumors (*p* < 0.0001), as shown in Figure 4.

#### 2.3.2. Association with Intravesical Recurrence and Progression

**Figure 5:**
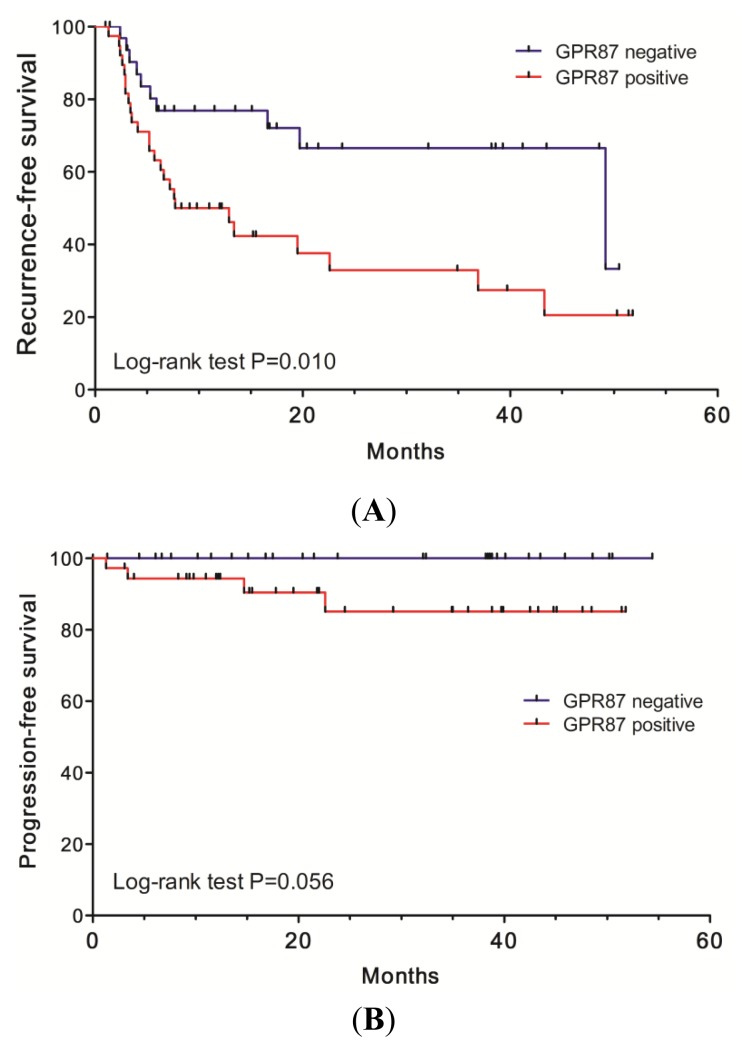
Intravesical recurrence-free survival curves (**A**) and progression-free survival curves (**B**) Blue line: patients with GPR87-negative bladder cancers, Red line: patients with GPR87-positive bladder cancers.

Intravesical recurrence-free survival was analyzed with a median follow-up period of 9.8 months (1.0–51.8 months) in relation to GPR87 expression as shown in Figure 5A. Patients with GPR87-negative tumors had longer intravesical recurrence-free survival periods than those with GPR87-positive tumors (*p* = 0.010). The two-year recurrence-free rate in GPR87-negative tumors was 67%, while that in GPR87-positive tumors was 33%. We performed univariate analysis to investigate the relationship of intravesical recurrence with 10 clinical parameters, including GPR87 staining status, and found that multiplicity, tumor size and GPR87 staining status correlated with recurrence (Table 1). Multivariate analysis revealed that only GPR87 staining status was an independent prognostic parameter for intravesical recurrence in this cohort as shown in Table 1 (*p* = 0.041).

Figure 5B shows progression-free survival curves of both GPR87-positive and GPR87-negative tumor groups. Progression from non-muscle-invasive tumor to muscle invasive or metastatic tumor was more frequently observed in patients with GPR87-positive tumors, although it did not reach statistical significance (*p* = 0.056).

In the present study, elevated expression of GPR87 mRNA was found in five of seven human bladder cancer cell lines. In addition, positive staining of GPR87 was observed in 54% of non-muscle invasive bladder cancer samples while no positive staining was found in adjacent normal bladder mucosa. These findings are in line with the previous study using gene tip technology [10]. Suppression of GPR87 gene expression in a human bladder cancer cell line, HT1197, which overexpressed GPR87, significantly reduced cellular viability. We identified a similar gene silencing effect in other bladder cancer cell lines and one prostate cancer cell line in the transfection experiment with adenovirus expressing shRNA targeting GPR87 (data not shown). Although we have not examined cell lines other than bladder and prostate cancers, these results suggest that GPR87-mediated signal transduction plays a pivotal role in the cellular proliferation of many solid cancers, including lung, cervix and head and neck cancers, in which GPR87 overexpression has been reported [9,10].

Surprisingly, 15 years before the present study, a German research group reported that a human bladder cancer cell line, J82, expresses various G protein-coupled receptors. They also found that LPA stimulated J82 cell migration. LPA has been detected in human body fluids, including sera and urine [11]. Lysophospholipase D (lysoPLD) catalyzes lysophosphatidylcholine to produce LPA, and lysoPLD has been detected in urine. Furthermore, LPA was detected in urine samples from proteinuria patients in concentrations ranging from 0.02 to 0.86 μM [11] and has also been electrophysiologically identified in serum [12]. These results indicate that LPA in urine and serum can stimulate urothelial carcinoma cells of the bladder via GPR87 signal transduction.

One clinical obstacle in non-muscle-invasive bladder cancer is its frequent recurrence inside of the bladder and sometimes in the upper urinary tracts and the urethra. Up to 50% of non-muscle-invasive bladder cancers recur within 2 years after transurethral resection of all visible tumors [13,14]. Patients who have been treated for non-muscle-invasive bladder cancer should undergo periodical cystoscopic examination (every 3 months for 2 years and every 4–6 months thereafter), which undoubtedly negatively impacts patient quality of life. Several clinical and pathological features, including the size and number of tumors at diagnosis, tumor grade and positive urine cytology, have been reported to be risk factors associated with recurrence. Prediction by such clinicopathological features, however, remains imperfect, and new markers are needed [15–18]. The strong association of GPR87 expression with bladder cancer cell proliferation observed in the present *in vitro* experiments may explain the unfavorable recurrence-free survival curve in GPR87-positive patients. Univariate analysis demonstrated that GPR87 protein expression, tumor multiplicity and maximal tumor diameter correlated with intravesical recurrence, while multivariate analysis revealed that GPR87 expression was the sole independent predictor for recurrence. However, these results should be interpreted in the context of several limitations. First, this analysis was based on a retrospective cohort. Second, 27 (38%) of 71 patients had received previous transurethral treatment (GPR87 positive group: 12 of 38 patients *vs.* GPR87 negative group: 15 of 33 patients). Third, 31 (44%) of 71 patients underwent adjuvant intravesical instillation therapy (GPR87 positive group: 19 of 38 patients *vs.* GPR87 negative group: 12 of 33 patients). Despite these limitations, the striking correlation of GPR87 overexpression with intravesical recurrence described here indicates that a prospective clinical study to clarify the clinical significance of GPR87 as a predictive marker for recurrence in treatment-naïve cohorts is warranted.

Another clinical concern regarding non-muscle-invasive bladder cancer is progression to muscle-invasive cancer. Progression and/or metastasis of tumors is not frequent (5% to 10%), but once it occurs, it seriously affects survival. Currently available parameters for predicting progression are still unsatisfactory [15,16], and improved risk assessment for progression using new biomarkers is needed. We performed another analysis of GPR87 staining status using a cohort of patients with muscle-invasive bladder cancer and demonstrated that GPR87 was positive in 13 (87%) of 15 patients; moreover, all of these GPR87-positive tumors were high-grade cancers. We have not yet investigated the association of GPR87 expression with tumor invasion and metastasis *in vitro*. However, it is interesting that the progression-free survival curve in patients with GPR87-positive tumors was unfavorable compared to those with GPR87-negative tumors, although this finding was not quite significant. Association of LPA-mediated signaling with tumor progression in carcinomas other than urothelial carcinoma has been reported. In hepatocellular carcinoma, LPA stimulates cancer cell invasion in a LPA-receptor dependent manner [19]. In prostate carcinoma, increased LPA-dependent RHO signaling enhances tumor invasion [20]. We also have reported enhanced expression of autotaxin, LPA-producing enzyme, in prostate cancers was correlated with tumor grade and capsular invasion [21]. These results suggest that GPR87 is a potential prognostic marker for the progression of non-muscle-invasive bladder cancers.

Urothelial cancers respond to platinum-based combination chemotherapy. The efficacy of this treatment, however, is limited, and the prognosis of metastatic bladder cancer is still very poor [22]. From a therapeutic viewpoint, GPR87 seems to be a promising molecular target. The results of the present study indicate that GPR87 is deeply involved in tumor recurrence and progression.

## 3. Experimental Section

### 3.1. Cell Lines

Five human bladder cancer cell lines (HT1197, J253, J82, RT112 and T24) were obtained from ATCC^®^ through the official Japanese distributor. RT4 and TCCSUP were kindly provided by Prof. Margaret Knowles, University of Leeds, UK. These cells were subjected to quantitative RT-PCR analyses and the silencing experiment, as described below. All cells were maintained in RPMI 1640 medium (Sigma-Aldrich, St. Louis, MO, USA) supplemented with 10% fetal bovine serum at 37 °C in a 5% CO_2_ atmosphere.

### 3.2. RNA Preparation and Real-Time RT-PCR

Total RNA was extracted from bladder cancer cells using TRIzol RNA isolation reagent (Life Technologies, Carlsbad, CA, USA). cDNA was synthesized using TaqMan reverse transcriptase (Applied Biosystems, Foster City, CA, USA). Real-time quantitative RT-PCR was performed with the StepOnePlus Real-Time PCR System (Applied Biosystems). The primers and probes were purchased from the Assays-on Demand Gene Expression Assay Mix (GPR87 ID Hs00225057_m1, Applied Biosystems). Each sample was assayed in triplicate. The comparative threshold cycle method was used to calculate gene expression in each sample relative to the value in these bladder cancer cells using GAPDH (ID Hs99999905_m1, Applied Biosystems) as a control for normalization among samples.

### 3.3. Protein Preparation and Western Blots

Whole-cell lysate was prepared from each cell line in Cell Lysis Buffer M (Wako Pure Chemical Industries, Ltd., Osaka, Japan), according to the manufacturer’s instructions. The protein concentrations were measured using Protein Assay (Bio-Rad, Laboratories, Inc., Hercules, CA, USA). Protein samples were separated in 10% Mini-PROTEAN Precast Gel (Bio-Rad, Laboratories, Inc., Hercules, CA, USA) and transferred to Amersham Hybond ECL Nitrocellulose membranes (GE Healthcare, Buckinghamshire, UK). A primary antibody against human GPR87 (#G358, Assay Biotechnology Company, Sunnyvale, CA, USA) was used at a dilution of 1:1000. A signal corresponding to GPR87 was detected using an ECL Advanced Western Blotting Detection Kit (GE Healthcare, Buckinghamshire, UK). A monoclonal antibody against ß-actin (clone AC74, Sigma, St. Louis, MO, USA) (dilution 1:5000) was used to control sample loading. The ß-actin signal was also detected with the chemiluminescence kit (GE Healthcare, Buckinghamshire, UK).

### 3.4. Ad-shGPR87 Mediated Knockdown

Three siRNA oligonucleotides targeting GPR87 were designed using the siRNA Design Support System (Takara Bio Inc., Otsu, Japan). The sense strand sequences were: GPR87-siRNA-A, 5′-GUAAGGGAGAUACCUACCUTT-3′; GPR87-siRNA-B, 5′-CAAACCAGGAAUAACCUAU-3′; and GPR87-siRNA-C, 5′-GAAUCGAUAUGUACAAAGUTT-3′; GPR87-siRNA-Negat, 5′-UCUUAAUCGCGUAUAAGGCTT-3′. The siRNA transfection was performed in with TransIT-TKO transfection Reagents (Mirus, Madison, WI, USA) for efficient down-regulation of hGPR87 mRNA. Thereafter, the most efficient sequences, GPR87-siRNA-C, was selected for constructing shRNA adenoviral vectors. The shRNA template [forward strand: 5′-GPR87-siRNA-A sense strand + loop (TAGTGCTCCTGGTTG) + GPR87-siRNA-C antisense strand + polymerase III terminator (TTTTTT)] was synthesized. To produce a plasmid vector expressing shRNA (plasmid-shGPR87), this shRNA template was cloned into a pBAsi-hU6 plasmid vector (Takara Bio Inc., Otsu, Japan). An adenoviral vector was constructed using an Adenovirus Expression Vector Kit (Takara Bio Inc., Otsu, Japan). The insert with the human RNA polymerase III-dependent U6 promoter and shRNA template was produced from plasmid-shGPR87 using restriction enzyme digestion by EcoRV and it was then ligated into a pAxcwit2 cosmid vector. A replication-deficient recombinant adenoviral vector expressing shRNA targeting GPR87 under the control of the human U6 promoter (Ad-shGPR87) was constructed using the COS-TPC method. A control adenoviral vector expressing shRNA against the scramble sequence of GPR87-siRNA was also constructed (Ad-scramble). As to the transfection of Ad-shRNAs, 1 × 10^5^ cells were plated in a 6-well tissue culture plate and cultured for 24 h to yield 70%–80% confluence. Ad-shRNAs were transfected at a concentrate of 10 and 20 MOI for 1 h in incubator. The cells were analyzed 120 h after siRNA transfection for gene expression and cellular proliferation rates.

### 3.5. Cell Proliferation

Cells were seeded in 96-well plates at a concentration of 4000 cells/well. After the cells were transfected with Ad-shRNA, cell viability was determined by a 3-(4,5-dimethylthiazol-2-yl)-2, 5-diphenyltetrazolium bromide (MTT) assay using Cell Proliferation Kit I (Roche, Mannheim, Germany). The cells were incubated with 10 μL of MTT labeling reagent for 4 h and were then incubated with 100 μL of solubilization solution overnight. Finally, the cell viability in each well was measured in terms of optical density at a wavelength of 570 nm, with 750 nm as the reference wavelength. Each cell viability assay was performed in triplicate.

### 3.6. Patients and Tissue Specimens

Bladder tumor specimens were obtained by transurethral resection from 71 patients with non-muscle-invasive bladder cancer between January 2006 and December 2009. Prior to sample collection, written informed consent was obtained from all patients. The study protocol was approved by the institutional review board of Kagawa University Faculty of Medicine. No patient had concurrent or precedent tumors in the upper urinary tracts. All tumors examined were histologically confirmed to be urothelial carcinoma of the bladder. Clinical and pathological characteristics are shown in Table 2. Tissue specimens were fixed with 10% buffered formalin and embedded in paraffin until the immunohistochemical analyses. Tumor grades were described according to the 2004 WHO/ISUP classification system, and tumor stages were described according to the UICC TNM-classification, version 7 (2009).

### 3.7. Immunohistochemistry

The formalin-fixed paraffin-embedded tissue specimens were cut into several 4-μm sections and mounted on poly-l-lysine-coated slides. Sections were deparaffinized and rehydrated. One section of each specimen was subjected to hematoxylin and eosin staining for tumor grade assessment. The remaining slides were then heated in a pascal pressure chamber for 40 min in DAKO Target retrieval solution at pH 9.0 (DAKO, Glostrup, Denmark), and cooled to room temperature. After quenching endogenous peroxidase activity with DAKO REAL Peroxidase-Blocking Solution (DAKO, Glostrup, Denmark) for 10 min, the sections were treated for 1 h at room temperature with 5% bovine serum albumin. For GPR87 immunostaining, the slides were incubated for 45 min at room temperature with a rabbit polyclonal antibody against human GPR87/GPR95, LS-A1584 (MBL, Nagoya, Japan) at a dilution of 1:100. For determination of the Ki-67 index, the slides were incubated with a mouse monoclonal antibody against the Ki-67 antigen, M7240, (DAKO, Glostrup, Denmark) for 30 min at a dilution of 1:100. The sections were then incubated for 1 h with DAKO Envision Dual Link System-HRP (DAKO, Glostrup, Denmark). Antibody binding was visualized with 3,3′-diaminobenzidine tetrahydrochloride. Lastly, the sections were lightly counterstained with Mayer’s hematoxylin.

The immunostaining status of tumor foci was evaluated by a surgical pathologist (S.S.) who was blind to the clinical data. Because our preliminary experiments revealed that GPR87 expression was consistently negative in normal bladder mucosa, we used normal bladder mucosa as a negative control. HT-1197 human bladder cancer cells were fixed on slides and used as a positive control. GPR87 staining intensity was graded as “negative” or “positive”. The Ki-67 labeling index was determined by the average percentage of Ki-67-positive cancer cells per all cancer cells in 3 different 400-fold magnified fields. The cut-off value for Ki-67 positivity was set as 18% [23].

### 3.8. Statistical Analysis

Non-parametric tests were used to compare mean GPR mRNA levels and cellular viability measurements. Pearson’s chi-square test or Fisher’s exact test was used to assess significant differences in categorical variables. Differences in the Ki-67 labeling index in relation to GPR87 staining status was assessed with the Mann-Whitney rank sum test. Recurrence-free survival and progression-free survival were determined using the Kaplan-Meier method, and the resultant curves were compared with the log-rank test. The Cox proportional hazards model was used for multivariate analyses. A *p*-value less than 0.05 was considered to be statistically significant for all tests. All statistical analyses were carried out with the Statistical Package for Social Sciences software, version 16 (SPSS, Chicago, IL, USA).

## 4. Conclusions

Up-regulation of GPR87 is closely associated with intravesical recurrence and is possibly involved in the progression of non-muscle invasive bladder cancers. Further investigation to clarify the clinical importance of GPR87 as a predictive marker and a therapeutic target for bladder cancers is warranted.

## Figures and Tables

**Table 1:** Univariate and multivariate analyses of factors associated with intravesical recurrence-free survival.

Variables	Univariate analysis			Multivariate analysis		
	
	Hazard ratio	95% CI	*p*	Hazard ratio	95% CI	*p*
Gender (M *vs*. F)	1.55	0.690–3.486	0.289			
Onset (primary *vs*. recurrence)	1.17	0.587–2.345	0.652			
Multiplicity (single *vs*. multiple)	2.41	1.211–4.811	0.012	1.87	0.873–4.911	0.107
Maximal tumor size (<3 cm *vs*. ≥3 cm)	3.16	1.096–9.090	0.033	1.53	0.638–3.689	0.339
Tumor type (papillary *vs*. non-papillary)	1.45	0.574–3.663	0.432			
T stage (Ta *vs*. T1)	1.54	0.661–3.585	0.318			
Grade (low grade *vs*. high grade)	1.21	0.585–2.489	0.610			
Adjuvant intravesical chemotherapy	1.21	0.614–2.381	0.582			
Ki-67 (<18% *vs*. ≥18%)	1.61	0.555–2.426	0.691			
GPR87 (negative *vs*. positive)	2.40	1.230–4.666	0.010	2.25	1.035–4.911	0.041

**Table 2:** Characteristics of patients with non-muscle invasive cancer.

Parameters		
Age	range, median	41–95, 72

Gender	M	59
	F	12

Stage	Tis	2
	Ta	53
	T1	16

Tumor grade (2004WHO/ISUP)	Low grade	46
	High grade	25

Multiplicity of tumor	Single	34
	Multiple	37

Tumor type	papillary	58
	non-papillary	13

Maxmal tumor size	<3 cm	54
	≥3 cm	12
	unknown	5

On-set	Primary	44
	Recurrence	27

Adjuvant intravesical chemotherapy	Yes	31
	No	40

Ki-67 index	<18%	50
	≥18%	21

GPR87	positive	38
	negative	33

## Abbreviations

GPR87: G protein-coupled receptor 87
GPCR: G protein-coupled receptor
LPA: lysophosphatidic acid
RT-PCR: reverse-transcription polymerase chain reaction
lysoPLD: lysophospholipase D
MTT: 3-(4,5-dimethylthiazol-2-yl)-2,5-diphenyltetrazolium bromide
shRNA: short hairpin RNA
cDNA: complementary DNA
GAPDH: glyceraldehyde 3-phosphate dehydrogenase
MOI: multiplicity of infection
